# Metabolic engineering of *Escherichia coli* for l-malate production anaerobically

**DOI:** 10.1186/s12934-020-01422-0

**Published:** 2020-08-18

**Authors:** Youming Jiang, Tianwen Zheng, Xiaohan Ye, Fengxue Xin, Wenming Zhang, Weiliang Dong, Jiangfeng Ma, Min Jiang

**Affiliations:** State Key Laboratory of Materials-Oriented Chemical Engineering, College of Biotechnology and Pharmaceutical Engineering, Jiangsu National Synergetic Innovation Center for Advanced Materials (SICAM), Nanjing Tech University, Nanjing, 211816 People’s Republic of China

**Keywords:** l-malate, *Escherichia coli*, Anaerobic fermentation, ATP, NAD(H)

## Abstract

**Background:**

l-malate is one of the most important platform chemicals widely used in food, metal cleaning, textile finishing, pharmaceuticals, and synthesis of various fine chemicals. Recently, the development of biotechnological routes to produce l-malate from renewable resources has attracted significant attention.

**Results:**

A potential l-malate producing strain *E. coli* BA040 was obtained by inactivating the genes of *fumB*, *frdABCD*, *ldhA* and *pflB*. After co-overexpression of *mdh* and *pck*, BA063 achieved 18 g/L glucose consumption, leading to an increase in l-malate titer and yield of 13.14 g/L and 0.73 g/g, respectively. Meantime, NADH/NAD^+^ ratio decreased to 0.72 with the total NAD(H) of 38.85 µmol/g DCW, and ATP concentration reached 715.79 nmol/g DCW. During fermentation in 5L fermentor with BA063, 41.50 g/L glucose was consumed within 67 h with the final l-malate concentration and yield of 28.50 g/L, 0.69 g/g when heterologous CO_2_ source was supplied.

**Conclusions:**

The availability of NAD(H) was correlated positively with the glucose utilization rate and cellular metabolism capacities, and lower NADH/NAD^+^ ratio was beneficial for the accumulation of l-malate under anaerobic conditions. Enhanced ATP level could significantly enlarge the intracellular NAD(H) pool under anaerobic condition. Moreover, there might be an inflection point, that is, the increase of NAD(H) pool before the inflection point is followed by the improvement of metabolic performance, while the increase of NAD(H) pool after the inflection point has no significant impacts and NADH/NAD^+^ ratio would dominate the metabolic flux. This study is a typical case of anaerobic organic acid fermentation, and demonstrated that ATP level, NAD(H) pool and NADH/NAD^+^ ratio are three important regulatory parameters during the anaerobic production of l-malate.

## Background

l-malate is one of the intermediates in the tricarboxylic acid (TCA) cycle, and it has been extensively used in beverage, chemical, agricultural, food and pharmaceutical industries. Considering its wide applications, l-malate has been identified as one of the twelve most promising platform chemicals that can be produced from biomass by the US Department of Energy [1]. Currently, the main route for commercial production of l-malate is by enzymatic synthesis from fumarate using free/immobilized fumarase or whole cells with high fumarase activity [2, 3]. However, as the substrate fumarate is largely derived from unsustainable fossil resource, the development of biotechnological routes to produce l-malate from renewable biomass has attracted significant attention recently [4].

In the last decades, a variety of microorganisms have been exploited and developed for the production of l-malate by a variety of rational and irrational design with the breeding techniques and genetic strategies [5]. *Escherichia coli*, as an ideal microbial cell factory, could be developed for the production of l-malate. Up to now, four major l-malate biosynthesis pathways have been identified in *E. coli*: (1) Reductive TCA (rTCA) pathway. This pathway has a net fixation of one mole CO_2_ per mole l-malate, resulting in a maximum theoretical l-malate yield of 2 mol/mol glucose [6]. (2) One-step carboxylation route with specific malic enzymes. Even though this pathway has the same maximum theoretical yield, it is difficult to achieve as the malic enzymes having higher affinity towards l-malate to pyruvate [7]. (3) Oxidative TCA pathway. This pathway normally occurrs in the mitochondrial or cytoplasmic TCA cycle aerobically with the condensation of citric acid and acetyl-CoA via the oxidative TCA branch. As CO_2_ is released in this process, the maximum theoretical yield is limited to 1 mol/mol glucose [8]. (4) Glyoxylate shunt route. This pathway has the maximum theoretical yield of 1 mol/mol, and occurs under aerobic condition or by genetic regulation [9].

**Fig. 1:**
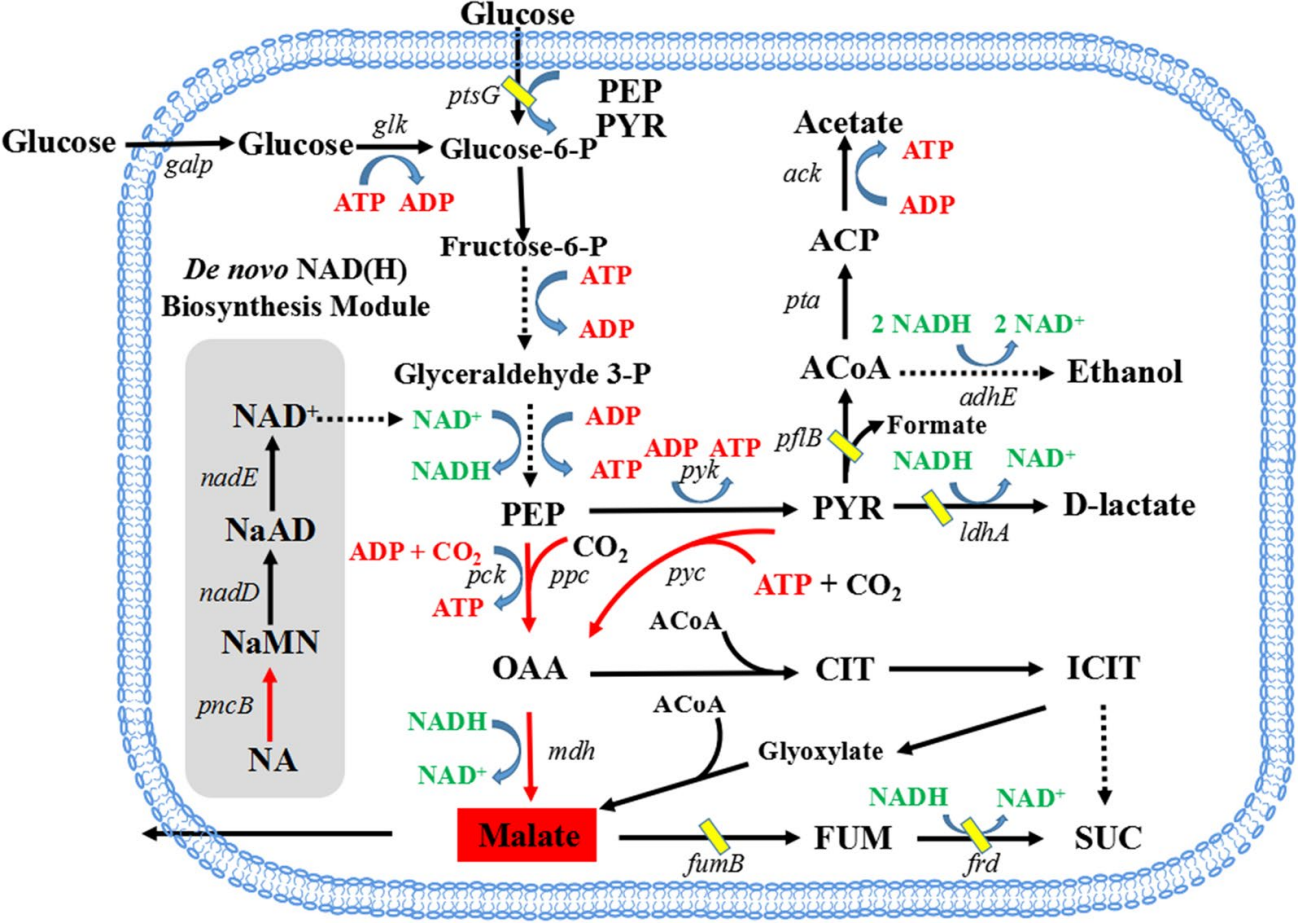
Engineered metabolic network for L-malate production from glucose in *E. coli* anaerobically. The undesired genes deleted in this study are marked in the pathways are deleted as marked with yellow oblique bar. The over-expressed and heterologous introduction pathways were marked with red bold lines. Not all enzyme-catalyzed steps and intermediates are shown. Pyr, pyruvate; Fum, Fumarate; Suc, succinate; CIT, citrate; ICIT, isocitrate; PEP, phosphoenolpyruvate; OAA, oxaloacetate; ACoA, acetyl-coenzyme A; ACP, acetyl phosphate; NA, nicotinic acid; NaMN, nicotinic acid mononucleotide; NAD, nicotinamide adenine dinucleotide; NaAD, desamido NAD. The genes involved in the metabolic pathways: *ldhA*, lactate dehydrogenase; *pflB*, pyruvate-formate lyase; *frdABCD*, fumarate reductase; *fumB*, fumarase; *mdh*, malate dehydrogenase; *ppc*, phosphoenolpyruvate carboxylase; *pck*, phosphoenolpyruvate carboxykinase; *pykF*, *pykA*, pyruvate kinase; *pta*, phosphate acetyltransferase; *ackA*, acetate kinase; *adhE*, alcohol dehydrogenase; *ptsG*, phosphotransferase system; *pncB*, NA phosphoribosyltransferase; *nadD*, NaMN adenylyltransferase; *nadE*, NAD synthetase

Among these pathways, the rTCA route is recognized as the most efficient route, which has the highest maximum theoretical yield of and higher catalytic efficiency [10]. As shown in Fig. 1, C3 intermediates including phosphoenolpyruvate (PEP) and pyruvate to oxaloacetate (OAA) are key steps to synthesis of l-malate by the rTCA route. Zhang et al. [11] have overexpressed PEP carboxykinase in *E. coli* WGS-10 mutant, which resulted in the accumulation of 9.25 g/L l-malate. Jantama et al. [12] constructed recombinant *E. coli* KJ071 which accumulated 516 mM l-malate with a yield of 1.4 mol/mol. PEP carboxylase has also been overexpressed in *E. coli* to enhance l-malate accumulation [13]. A similar *E. coli* KJ073 mutant produced 34 g/L l-malate with 1.42 mol/mol yield by dual-phase fermentation [6].

In addition to regulation of the carbon metabolic pathway related enzymes, many studies have demonstrated that the intracellular NAD(H) availability and cellular redox state significantly affected the metabolic flux distribution and metabolite levels, and many researchers have investigated the effects of cofactors on the generation of target products [14, 15]. Under anaerobic conditions, mixed-acid fermentation was carried out to maintain its homeostatic redox balance in wild-type *E. coli* [16]. In order to accumulate l-malate, several NADH-consuming pathways should be blocked, and oxaloacetate reduction should be the primary route for the regeneration of NAD^+^. Consequently, we assumed that the limited NAD^+^ regeneration ability might lead to serious decline in cell growth and substrate utilization in mutant *E. coli*.

In this study, we aim to construct engineered *E. coli* to produce l-malate anaerobically with CO_2_ fixation. Overexpression of native *mdh* gene and *pncB* gene or reduction of the glycolysis rate by inactivating the *ptsG* gene were carried out to investigate the effects on intracellular NAD(H) pool, NADH/NAD^+^ ratio and malate accumulation. Then, three carboxylation enzymes were overexpressed to improve the carboxylation capacity of C3 intermediates and redirecting more carbon flux into malate biosynthesis pathway. Furthermore, multiple strategies were combined and compared by co-overexpression of related genes, and then the relationships among the intracellular NAD(H), NADH/NAD^+^ ratio, ATP supply, carboxylation efficiency, and l-malate production were systemically studied.

## Results and discussion

### Engineering the *E. coli* host strain to accumulate l-malate anaerobically

As an important intermediate of TCA cycle, l-malate does not naturally accumulate in *E. coli*. In order to make the strain to produce l-malate, the enzymes including fumarase (encoded by *fumB*) and fumarate reductase (encoded by *frdABCD*) were deleted in a *ldhA* and *pflB* double mutant strain *E. coli* BA002. As a result, a potential l-malate producing strain *E. coli* BA040 was obtained.

To investigate the fermentation phenotype of the mutants, fermentations with BA040 were carried out in sealed serum bottles under dual-phase conditions. As shown in Table 1, BA040 consumed 8.5 g/L glucose and produced 1.73 g/L l-malate with pyruvate and succinate as main by-products after 48 h fermentation. Glucose consumption rate and metabolic ability of BA040 were very low under the anaerobic conditions, suggesting that there might be some potential metabolic obstacles after inactivating *fumB* and *frdABCD*. It has been reported that after eliminating the NADH-dependent pathways in *E. coli*, the mutant strains cannot utilize glucose anaerobically due to the limited ability of NAD^+^ regeneration [17]. As shown in Table 2, the ratio of NADH/NAD^+^ was relative high, which was consistent with the literature. Thus, the balance between NADH oxidation and regeneration should be regulated to improve the l-malate production.

**Table 1:** Results of dual-phase fermentations of the recombinant strains in sealed bottles after 48 h

Strain^*a*^	Consumed glucose (g/L)	Fermentation product titer (g/L)			Malate yield^b^ (g/g)
		Pyruvate	Succinate	Malate	
BA040	8.50 ± 0.24	0.97 ± 0.02	1.23 ± 0.02	1.73 ± 0.04	0.20 ± 0.01
BA041	10.50 ± 0.42	1.85 ± 0.08	2.46 ± 0.05	3.53 ± 0.25	0.34 ± 0.01
BA042	14.50 ± 0.20	2.52 ± 0.15	1.87 ± 0.08	4.39 ± 0.12	0.30 ± 0.01
BA050	9.50 ± 0.50	1.81 ± 0.21	1.58 ± 0.01	3.22 ± 0.36	0.34 ± 0.01
BA051	11.50 ± 0.50	1.69 ± 0.50	1.46 ± 0.02	5.30 ± 0.50	0.46 ± 0.50
BA052	10.00 ± 0.45	0.58 ± 0.01	1.85 ± 0.05	4.39 ± 0.21	0.44 ± 0.01
BA061	15.00 ± 0.42	2.87 ± 0.22	1.79 ± 0.02	9.39 ± 0.42	0.63 ± 0.01
BA062	15.00 ± 0.55	2.89 ± 0.14	1.45 ± 0.01	9.19 ± 0.55	0.61 ± 0.01
BA063	18.00 ± 0.46	2.67 ± 0.15	1.25 ± 0.02	13.14 ± 0.35	0.73 ± 0.01

Fermentations were carried out in a 100 mL sealed bottle with 30 ml LB medium with 30 g/L glucose and 16 g/L magnesium carbonate hydroxide (37 °C, 170 rpm). Anaerobiosis was achieved during growth with added bicarbonate to ensure an atmosphere of CO_2_

^*a*^All the strains were tested with a two-stagedual-phase process (aerobic cell growth and anaerobic L-malate production), average fermentation characteristics from triplicate sealed bottle fermentations are shown

^*b*^ Yield was calculated as grams of L-malate produced per gram of glucose consumed

**Table 2:** Intracellular NAD(H) and ATP concentration during in anaerobic phase at 48 h

Strain	NADH (µmol/g DCW^*a*^)	NAD^+^ (µmol/g DCW)	Total NAD(H) (µmol/g DCW)	NADH/NAD^+^	ATP (nmol/g DCW)
BA040	6.16 ± 0.12	9.47 ± 0.20	15.63 ± 0.34	0.65 ± 0.02	338.89 ± 10.84
BA041	5.93 ± 0.41	11.57 ± 0.11	17.50 ± 0.17	0.51 ± 0.01	362.45 ± 12.55
BA042	10.75 ± 0.62	17.65 ± 0.55	28.40 ± 0.42	0.61 ± 0.01	388.85 ± 16.64
BA050	4.85 ± 0.22	9.14 ± 0.28	13.99 ± 0.65	0.53 ± 0.01	358.84 ± 15.82
BA051	5.85 ± 0.05	10.86 ± 0.25	16.71 ± 0.15	0.53 ± 0.02	385.65 ± 18.85
BA052	5.93 ± 0.01	11.57 ± 0.11	17.50 ± 0.16	0.51 ± 0.01	354.25 ± 12.46
BA061	14.04 ± 0.74	16.73 ± 0.86	30.77 ± 0.53	0.84 ± 0.02	667.43 ± 23.87
BA062	17.71 ± 0.43	20.56 ± 0.32	38.27 ± 0.93	0.86 ± 0.03	683.26 ± 27.55
BA063	16.33 ± 0.51	22.52 ± 0.52	38.85 ± 0.37	0.72 ± 0.02	715.79 ± 25.78

Each value is the mean of three parallel replicates ± standard deviation

^*a*^ DCW, dry cell weight

### Comparing three NAD(H/+) regulation strategies to improve l-malate production

Here, we conducted three NAD(H/+) regulation strategies: (1) Enhance the NADH oxidation by overexpression of malate dehydrogenase (encoded by *mdh*). (2) Enlarge the NAD(H/+) pool size to enhance the cellular robustness by overexpression of nicotinic acid phosphoribosyltransferase (encoded by *pncB*). (3) Reduce the glycolysis efficiency to decrease NADH synthesis by deletion of *ptsG* gene encoding the glucose-specific permease EIICB^glc^.

Both *mdh* and *pncB* were overexpressed using the pTrc99a plasmid. As shown in Table 3, the specific activity of MDH in BA041 was 72.80 U/mg which was 19-fold higher than that in BA040 (3.80 U/mg), and the specific activity of NAPRTase in BA042 was 19.45 U/mg which was almost tenfold higher than that in BA040 (1.94 U/mg), demonstrating that MDH and NAPRTase were significantly overexpressed in BA041 and BA042, respectively. Besides overexpression, *ptsG* gene of BA040 was deleted by λ Red homologous recombination to obtain BA050.

**Table 3:** Determination of specific activities of MDH, NAPRTase, PPC, PYC and PCK in crude extracts of the control and the recombinant strains

Strain	MDH activity (U/mg)	NAPRTase activity (U/mg)	PPC activity (U/mg)	PYC activity (U/mg)	PCK activity (U/mg)
BA040	3.80 ± 0.07	1.94 ± 0.04	2.24 ± 0.12	ND^*a*^	0.03 ± 0.01
BA041	72.80 ± 0.06	2.05 ± 0.05	2.26 ± 0.05	ND	0.01 ± 0.01
BA042	4.80 ± 0.08	19.45 ± 0.25	3.11 ± 0.05	ND	0.02 ± 0.01
BA050	3.50 ± 0.07	0.10 ± 0.01	2.43 ± 0.04	ND	0.02 ± 0.01
BA051	3.63 ± 0.03	1.49 ± 0.02	20.03 ± 0.23	ND	0.01 ± 0.01
BA052	4.50 ± 0.05	1.47 ± 0.03	3.02 ± 0.15	1.82 ± 0.14	0.01 ± 0.01
BA061	5.20 ± 0.05	1.50 ± 0.02	ND	ND	1.94 ± 0.06
BA062	4.80 ± 0.12	18.74 ± 0.35	ND	ND	2.15 ± 0.05
BA063	71.70 ± 2.50	1.80 ± 0.04	ND	ND	2.24 ± 0.08

Each value is the mean of three parallel replicates ± standard deviation

^*a*^ND, not detected

Fermentations with these three recombinants were also carried out in sealed serum bottles. As shown in Table 1, glucose consumption, l-malate production and yield in BA041 were increased by 23% (10.50 g/L), 104% (3.53 g/L) and 70% (0.34 g/g) compared to those of BA040. Also, a maximal l-malate production of 4.39 g/L was obtained by BA042 with the yield of 0.30 g/g. BA050 consumed 9.50 g/L glucose and accumulated 3.22 g/L l-malate with the yield 0.34 g/g, which were increased by 12%, 86% and 65% respectively compared with that of BA040. Thus, l-malate production of all recombinants were improved with any kind of NAD(H/+) regulation strategy.

Furthermore, NAD(H) assay results (Table 2) showed that total NAD(H) pool increased by 82% in *E. coli* BA042 (28.40 µmol/g) than that of BA040 (15.63 µmol/g), while NAD(H) pool of BA041 and BA050 had no significant difference from that in BA040. In particular, the ratio of NADH/NAD^+^ in *E. coli* BA041 and BA050 decreased to 0.51 and 0.53, compared to that of BA040 (0.65). There was no significant change of NADH/NAD^+^ ratio between BA042 and BA040. In conclusion, to enlarge the NAD(H) pool and/or to decrease NADH/NAD^+^ ratio in *E. coli* were both beneficial for the glucose consumption and l-malate production anaerobically.

### Effects of introducing/enhancing pathways of carboxylation on l-malate production

It is well recognized that carboxylation from PEP or pyruvate to OAA plays a significant role in high-efficiency synthesis of C4-dicarboxylic acids anaerobically. Generally, l-malate is primarily synthesized from PEP, which is converted to OAA and then to l-malate via the rTCA branch in *E. coli* strains (Fig. 1). Moreover, PCK is considered more appropriate than PPC for the production of C4-dicarboxylic acids under anaerobic conditions since PCK generates additional ATP during the process of PEP convert to OAA [18]. It was reported that a biotin-dependent PYC could catalyze the carboxylation of pyruvate to OAA [19]. Here, three carboxylation enzymes were introduced into *E. coli* BA050. A native *ppc* gene and a *pyc* gene from *Lactococcus lactis* were overexpressed in *E. coli* BA050 resulting in the recombinant strains BA051and BA052, respectively. A *pck* gene from *Bacillus subtilis* (*Bspck*) was heterogeneous overexpressed in the strain BA060 (*ppc* deleted) to obtain BA061 with PCK as the sole enzyme of PEP carboxylation.

The specific activities of PPC, PYC and PCK were assayed in various recombinants (shown in Table 3). The specific activity of PPC in strain BA051 was 20.03 U/mg, approximately eightfold higher than that of BA050 (2.43 U/mg). The absence of detectable PCK activity was consistent with the fact that PEP carboxylase played the major role for the carboxylation of PEP. The specific activity of PYC in BA052 was 1.82 U/mg and 1.94 U/mg activity of PCK was detected in *E. coli* BA061 while it was almost undetectable in other recombinants. The results showed that the native or exogenous enzymes were successfully overexpressed.

To select the most efficient strain for l-malate production, the dual-phase fermentation was carried out in sealed bottles with these three strains and the results are summarized in Table 1. All the recombinants (BA051, BA052, BA061) displayed increased metabolic efficiency compared with BA050. The carbon flux was successfully redirected from C3 intermediates to rTCA branch by the introduction of carboxylation pathways, leading to increased titer and yield of l-malate. Among them, BA061 achieved the highest concentration of l-malate (9.39 g/L) with a yield of 0.63 g/g, 191% and 85% higher than those of strain BA050. Moreover, glucose consumption was also increased to 15.00 g/L with a 58% enhancement compared to that of BA050. However, the concentration of pyruvate was increased from 1.88 g/L to 2.87 g/L in BA061. It might be attributed to the activated malic enzyme activities which catalyze the reversible oxidative decarboxylation of l-malate to pyruvate and CO_2_ [20].

In addition, the total NAD(H) in BA061 dramatically increased to 30.77 µmol/g DCW which was 2.2-fold higher than that of BA050, and NADH/NAD^+^ ratio increased from 0.53 in BA050 to 0.84 in BA061 (Table 2). It indicated that large enough NAD(H) pool could overcome the relative high NADH/NAD^+^ ratio and improve the glucose consumption and l-malate production. Another noteworthy observation was the enhanced intracellular ATP concentration in BA061 (667.43 nmol/g DCW), which was almost threefold of BA050 under anaerobic conditions. There were minor influences on the improvement of ATP levels by the overexpression of PPC and PYC. Accordingly, the results indicated that the enhanced ATP level could enlarge the intracellular NAD(H) pool under anaerobic condition.

### Combining NAD(H) regulation and overexpressing carboxylation enzymes to enhance l-malate production

As stated above, larger NAD(H) pool and lower NADH/NAD^+^ ratio are beneficial for glucose consumption and l-malate production. To investigate whether the l-malate production could be further enhanced by combining NAD(H) regulation and overexpressing carboxylation enzymes, *pncB* or *mdh* gene was co-overexpressed with *pck* in strain BA060 to obtain BA062 and BA063, respectively. The specific activities shown in Table 2 confirmed that all the genes were co-expressed successfully.

Dual-phase fermentations were carried out in sealed bottles to compare the fermentation performance of different recombinants and the results are summarized in Table 1. Even NAPRTase and PCK were functional expressed in BA062, l-malate titer (9.19 g/L) was slightly lower than that of the reference strain BA061 (9.39 g/L). On the other hand, a same level of glucose consumption (15 g/L) was achieved by BA062 compared with BA061, even with a higher intracellular NAD(H) in BA062 (38.27 µmol/g DCW). After co-overexpression of *mdh* and *pck*, BA063 achieved higher glucose consumption (18 g/L), leading to an increase in l-malate titer and yield of 13.14 g/L and 0.73 g/g, respectively. Meantime, NADH/NAD^+^ ratio decreased to 0.72 even though the total NAD(H) in BA063 was similar with that of BA061 (Table 2). The lower NADH/NAD^+^ ratio might be attributed to the increased MDH activity which further strengthened the metabolic flux from OAA toward l-malate and enhanced the regeneration of NAD^+^.

Accordingly, there might be an inflection point, that is, increasing NAD(H) availability would not further contribute to the utilization of glucose and the production of l-malate in *E. coli* strains when the intracellular total NAD(H) already meet the metabolic needs. And then, NADH/NAD^+^ ratio would dominate the metabolic flux distribution.

### Fermentation of recombinant strain in a 5-L fermenter

**Fig. 2:**
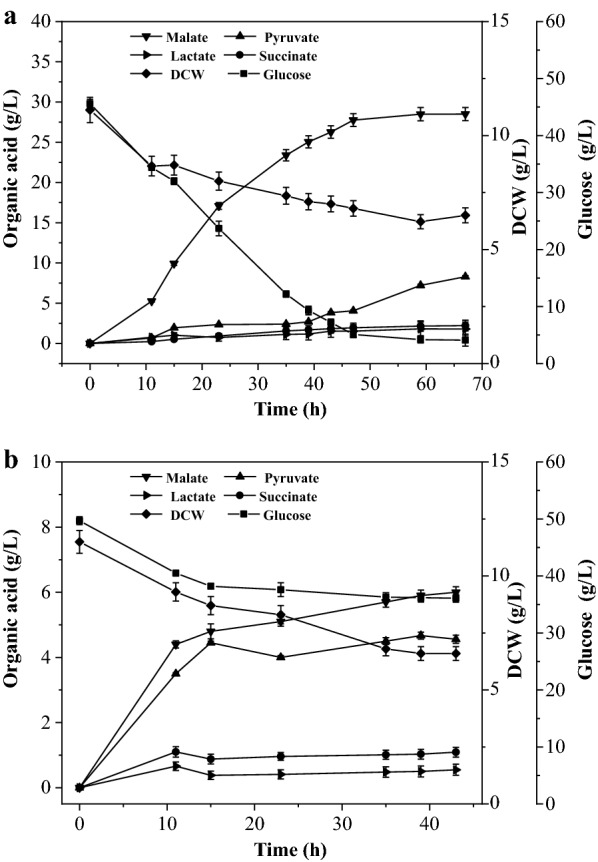
Anaerobic phase of recombinant strain BA063 with CO_2_ (**a**) and N_2_ (**b**) to maintain the anaerobic condition in 5-L fermenter. The experiments were performed in triplicate. DCW, Dry cell weight

To achieve high titer, dual-phase fermentations of the recombinant BA063 were carried out with defined minimal medium and glucose as carbon source (50 g/L initial glucose) in a 5-L fermenter. The time profiles of biomass, concentrations of glucose, and metabolites are presented in Fig. 2. Aerobic culture was finished when DCW reached around 11 g/L when nitrogen source was almost depleted, and then fermentations were shifted into anaerobic phase by sparging N_2_ or CO_2_. During the anaerobic phase with N_2_ (Fig. 2b), glucose consumption was inhibited and only 13.6 g/L glucose was consumed within 43 h. The major products were l-malate and pyruvate with the yields of 0.44 g/g and 0.34 g/g. When the anaerobic phase was supplied with heterologous CO_2_ (Fig. 2a), 41.50 g/L glucose was consumed within 67 h with the final l-malate concentration and yield of 28.50 g/L, 0.69 g/g. The DCW under either condition was obviously decreased at first 10 h and then almost keep constant until the end of the fermentation. We designed this no-growth anaerobic phase in order to minimize the carbon flux to biomass, and thus the l-malate yield could be enhanced.

The most surprising observation was that pyruvate was accumulated to a higher level (8.28 g/L) at the end of fermentation even supplying with heterologous CO_2_. It might be that the PCK activity decreased after a long cultivation or some malic enzymes were activated to catalyze malate to pyruvate. Moreover, lactate and succinate were accumulated as main by-products with the concentration reaching 1.78 g/L and 2.21 g/L, respectively. A possible reason for succinate accumulation might be caused by the activated glyoxylate shunt after 21.5 h of aerobic induction [21]. The unexpected accumulation of lactate suggested that there must be other pathways leading to lactate generation even though *ldhA* was inactivated in BA063. The yield of total organic acid including l-malate, pyruvate, succinate and lactate were 0.98 g/g and 0.89 g/g with and without CO_2_ supply. The increased total yield indicated that part of the CO_2_ source flew into organic acid, mainly l-malate and succinate, by carboxylation pathways from PEP and pyruvate to oxaloacetate.

## Conclusions

l-malate is one of the most important acidulants and flavor enhancers in beverage and food industries, and is also widely used in metal cleaning, textile finishing, pharmaceuticals, and synthesis of various fine chemicals. Here, we aim to construct engineered *E. coli* to produce l-malate anaerobically. We compared three NAD(H/+) regulation strategies and investigated the effects of introducing/enhancing pathways of carboxylation on l-malate production. From the results, we can draw the following conclusions: (1) The availability of NAD(H) was correlated positively with the glucose utilization rate and cellular metabolism capacities, and lower NADH/NAD^+^ ratio was beneficial to the accumulation of l-malate under anaerobic conditions. (2) Enhanced ATP level could significantly enlarge the intracellular NAD(H) pool under anaerobic condition. (3) There might be an inflection point, that is, the enhancement of NAD(H) pool before the inflection point is followed by the improvement of metabolic performance, while the increase of NAD(H) pool after the inflection point has no significant positive impacts and NADH/NAD^+^ ratio would dominate the metabolic flux distribution.

Pyruvate was around 2 g/L during the first 40 h anaerobic fermentation, and then began to accumulate in great quantities and the final pyruvate yield reached 0.20 g/g even supplying with heterologous CO_2_. Thus, we speculate PCK activity decreased after 40 h anaerobic cultivation or some malic enzymes were activated to catalyze malate to pyruvate. In future, we might inactivate the other malic enzymes and integrate *pck* and *mdh* genes into chromosomes to keep them sustained activity.

## Methods

### Strains and plasmids

All strains, plasmids, and primers used in this study are summarized in Table 4. All strains were stored in 20% (w/v) glycerol at − 80 °C. The malate dehydrogenase gene (*mdh*), phosphoenolpyruvate carboxylase gene (*ppc*) and nicotinic acid phosphoribosyltransferase gene (*pncB*) were amplified from the *E. coli* K12 genome, and phosphoenolpyruvate carboxykinase gene (*pck*) was amplified from *Bacillus subtilis* 168, and pyruvate carboxylase gene (*pyc*) was amplified from *Lactococcus lactis* subsp. cremoris NZ9000. The plasmids pTrc99a-*Bspck*, pTrc99a-*ppc*, pTrc99a-*pyc*, pTrc99a-*pncB* and pTrc99a-*mdh* were constructed previously in our laboratory [22–24].

**Table 4:** Strains and plasmids used in this study

Strains or plasmids	Relevant description	Sources
Strains		
BA002	*E. coli* K12, Δ*pflB*, Δ*ldhA*	Previous work
BA040	BA002, Δ*fumB*, Δ*frdABCD*	Previous work
BA041	BA040/pTrc99a-*mdh*	This study
BA042	BA040/pTrc99a-*pncB*	This study
BA050	BA040, Δ*ptsG*	This study
BA051	BA050/pTrc99a-*ppc*	This study
BA052	BA050/pTrc99a-*pyc*	This study
BA060	BA050, Δ*ppc*	This study
BA061	BA060/pTrc99a-*Bspck*	This study
BA062	BA060/pTrc99a-*Bspck*-*pncB*	This study
BA063	BA060/pTrc99a-*Bspck*-*mdh*	This study
Plasmids		
pMD19-T	T-vector; Ap^r^	TAKARA
pTrc99a	Expression vector with Trc promoter; Ap^r^	BioVector
pKD46	Vector containing Red (γ, β, and *exo*) recombination functions under control of P_araB_ promote, Ap^r^	CGSC
pTrc99a-*mdh*	*mdh* gene from *E. coli* K12	Previous work
pTrc99a-*pncB*	*pncB* gene from *E. coli* K12	Previous work
pTrc99a-*ppc*	*ppc* gene from *E. coli* K12	Previous work
pTrc99a-*pyc*	*pyc* gene from *Lactococcus lactis* subsp. cremoris NZ9000	Previous work
pTrc99a-*Bspck*	*pck* gene from *Bacillus subtilis* 168	Previous work
pTrc99a- *Bspck*-*mdh*	*pck* gene from *Bacillus subtilis* 168 and *mdh* gene from *E. coli* K12	This study
pTrc99a-*Bspck*-*pncB*	*pck* gene from *Bacillus subtilis* 168 and *pncB* gene from *E. coli* K12	This study

### Method for chromosomal deletions

Methods for chromosomal deletions using the classical *λ* Red homologous recombination [25] and gentamycin resistance gene removal have been described as manufacturer for the gene deletion of *E. coli* (RuiYang BioTech, Wuxi, China). Primers used for *ptsG* and *ppc* deletion are summarized in Additional file 1: Table S1.

### Media and fermentation conditions

LB medium contained the following constituents: Tryptone, 10 g/L; Yeast extract, 5 g/L; NaCl, 10 g/L.

The chemically defined (CD) fermentation medium contained: Citric Acid, 3.0 g/L; Na_2_HPO_4_∙7H_2_O, 3.0 g/L; KH_2_PO_4_, 8.00 g/L; (NH_4_)_2_HPO_4_, 8.00 g/L; NH_4_Cl, 0.20 g/L; (NH_4_)_2_SO_4_, 0.75 g/L; MgSO_4_∙7H_2_O, 1.00 g/L; CaCl_2_∙2H_2_O, 10.0 mg/L; ZnSO_4_∙7H_2_O, 0.5 mg/L; CuCl_2_∙2H_2_O, 0.25 mg/L; MnSO_4_∙H_2_O, 2.5 mg/L; CoCl_2_∙6H_2_O, 1.75 mg/L; H_3_BO_3_, 0.12 mg/L; Al_2_(SO_4_)_3_∙xH_2_O, 1.77 mg/L; Na_2_MoO_4_∙2H_2_O, 0.5 mg/L; Fe(III) citrate, 16.1 mg/L. The medium was supplemented with 2.0 mg/L biotin and 20.0 mg/L VB_1_. Ampicillin (100 mg/mL) and Gentamicin (30 mg/mL) were added when necessary.

As the poor anaerobic growth ability of *E. coli* BA040 and its derivants, dual-phase (aerobic growth phase is followed by anaerobic fermentation phase) fermentations were adopted in this study to investigate the production performance of engineered strains. During the fermentation with shake flasks, 1 mL of inoculum from an overnight LB culture was added to 100 mL fresh LB medium in a 500 mL flask bottle for aerobic growth at 37 °C and 200 rpm, and then the cells were induced at 30 °C and 170 rpm with the addition of 0.1 mM isopro-pyl-β-d-thiogalactopyranoside (IPTG) when the OD_600_ was reached to 0.7–1.0. After 8 h cultivation, cells were collected aseptically by centrifugation at 4 °C and 4100 rpm for 10 min. The harvested cells were resuspended in 100 mL sealed serum bottles containing 30 mL fresh LB medium supplemented with 30 g/L glucose, 24 g/L sterilized magnesium carbonate hydroxide to maintain the pH above 6.8. CO_2_ was pumped into the sealed serum bottles for at least 2 min with a sterilizing filter. The anaerobic cultures were incubated at 30 °C on a rotary shaker at 200 rpm for 48 h and all the samples were performed in three replicates.

For the fermentation carried out in 5-L fermenter, chemical defined medium was adopted. 2 mL of inoculum from an overnight LB culture was added to a 1000 mL flask containing 200 mL LB medium for aerobic growth at 37 °C and 200 rpm. After 6 h incubation, a 10% inoculum was transferred into the fermenter to start the dual-phase fermentation. 30 g/L glucose was added at the beginning of the aerobic phase at 30 °C and 0.1 mM IPTG was added to induce the cells after 4 h cultivation. The pH was controlled automatically at 6.8 by 2 M NaOH. After the residual glucose concentration was less than 5 g/L, the aerobic growth phase was terminated and directly transferred to anaerobic conditions (1) with the CO_2_ rate of 0.2 vvm and agitation of 200 rpm, and the pH was maintained automatically at 6.6 by 1 M Na_2_CO_3_ in the anaerobic stage. (2) with the N_2_ rate of 0.2 vvm and agitation of 200 rpm, and the pH was maintained automatically at 6.6 by 1 M NaOH in the anaerobic stage. Initial glucose concentration was adjusted to around 50 g/L.

### Cell biomass and residual glucose concentration

OD_600_ was analyzed using an ultraviolet–visible spectrophotometer (Spectrumlab 752S). Dry cell weight (DCW) was computed from a curve of optical density at 600 nm (OD_600_) to dry weight; an OD_600_ of 1.0 represents 400 mg dry weight per L. Glucose concentration was detected by a chronoamperometry method. In this detection process, a calibration line of current response vs. glucose concentration was first fitted by the continuous additions of a standard glucose concentration into a buffer solution. Then the target sample was added to calculate its glucose concentration by using the calibration line [26, 27].

### Metabolite analysis

Organic products were determined by high performance liquid chromatography (HPLC) (Chromeleon server monitor, P680 pump, Dionex, USA) with a UVD 170U ultraviolet detector at a wavelength of 215 nm and ion exchange chromatographic column (Aminex HPX-87H, 300 mm × 7.8 mm; BIO-RAD, USA). The flow rate was controlled at 0.5 mL/min with the mobile phase of 2.55 mM H_2_SO_4_. The yield (g/g) is defined as the ratio of accumulated organic acid (malate, pyruvate, et al.) to consumed glucose.

### Quantification of NAD^+^/NADH

The intracellular concentrations of NAD^+^ and NADH were assayed according to the method described previously [24].

### Measurement of intracellular ATP concentrations

To measure intracellular ATP concentrations, a BacTiter-GloTM Microbial Cell Viability assay kit on the GloMax^®^-Multi Detection System (Promega, Madison, WI, USA) was used. 1 mL of cold 30% (w/v) trichloroacetic acid was added to the samples (4 mL) and mixed thoroughly. Cells harvested at the each time were chilled immediately with liquid nitrogen for 5 min and then stored at − 80 °C for assay. Each of 100 µL sample was added to equivalent volume of reaction buffer and enzyme mixture and then thoroughly homogenized. The ATP concentration was calculated: ATP (nmol/g) = 10 × (0.9996 × log_10_Di-2.437)/DCW. The data Di is referred to the measured luminescence.

### Enzyme activity assays

Cells were harvested after 8 h induction by IPTG in the shake flasks by centrifugation at 12,000×*g* for 15 min and washed with 2 mL 100 mmol/L of Tris–HCl (pH 7.5) twice to determine the enzyme activities. The cells were subsequently resuspended in the same buffer and sonicated on ice for 10 min (a working period of 3 s in a 3 s interval for each cycle) at a power output of 300 W by using an ultrasonic disruptor (SCIRN72-IID, Ningbo, China). The cell debris was removed by centrifugation (12,000×*g* for 15 min at 4 °C), and the crude cell extracts were immediately used to determine the enzyme activities. The total protein concentration in the crude cell extracts was measured according to Bradford’s method with bovine serum albumin as the standard. To compare the specific activity of different samples, the same amount of protein was added for the measurement of the enzyme activity, and each measurement was performed in triplicate. The activities of PCK, MDH, PYC and NAPRTase were assayed as previously described [17, 23, 24, 28].

## Supplementary information

**Additional file 1: Table S1.** The primers used for plasmid construction and gene knockout.
