# Supplementary material for: Metabolic engineering of Escherichia coli for l-malate production anaerobically
Source: Microb Cell Fact. 2020 Aug 18;19:165. doi: 10.1186/s12934-020-01422-0 (PMC7437165; doi:10.1186/s12934-020-01422-0)
Supplement: Supplementary file 1 — Additional file 1: Table S1. The primers used for plasmid construction and gene knockout. [file 12934_2020_1422_MOESM1_ESM.docx]

**Table S1** The primers used for plasmid construction and gene knockout.

| **Name** | **Sequence (5’🡪3’)** | **Restriction site** |
| --- | --- | --- |
| *Bspck*-Frw | CACACAGGAAACAGA**CCATGG**ATGAACTCAGTTGATTTGACCGCTG | *Nco*Ⅰ |
| *Bspck*-Rev | GCCTGCAGGTCGAC**TCTAGA**TTATACGAGAGGGCCGCCTG | *Xba*Ⅰ |
| *mdh*-Frw | CACACAGGAAACAGA**CCATGGA**TGAAAGTCGCAGTCCTCGGC | *Nco*Ⅰ |
| *mdh*-Rev | GCCTGCAGGTCGAC**TCTAGA**TTACTTATTAACGAACTCTTCGCCCAGG | *Xba*Ⅰ |
| *pncB*-Frw | AGGAAACAGA**CCATGG**AATTCATGGATGACACAATTCGCTTCTCCTGTTC | *Nco*Ⅰ |
| *pncB*-Rev | GCCTGCAGGTCGAC**TCTAGA**TTAACTGGCTTTTTTAATATGCGGAAGG | *Xba*Ⅰ |
| *Bspck*-*mdh*-Frw | GGCCCTCTCGTATAA**TCTAGA**CAGCTTATCATCGACTGCAC | *Xba*Ⅰ |
| *Bspck*-*mdh*-Rev | TTGCATGCCTGCAG**GTCGAC**TTACTTATTAACGAACTCTTCGCC | *Sal*Ⅰ |
| *Bspck*-*pncB*-Frw | GGCCCTCTCGTATAA**TCTAGA**CAGCTTATCATCGACTGCAC | *Xba*Ⅰ |
| *Bspck*-*pncB*-Rev | TTGCATGCCTGCAG**GTCGAC**TTAACTGGCTTTTTTAATATGCGG | *Sal*Ⅰ |
| *ptsG* knockout | AAAGCACCCATACTCAGGAGCACTC |  |
|  | TGCCTTAGTCTCCCCAACGTCTTAC |  |
|  | GTTTACTACACCATCTTCCGCGTGC |  |
|  | AGATAATCCCTCCGAGTACGCCAGT |  |
| *ppc* knockout | GCATCTTATCCGACCTACACCTTTG |  |
|  | AGAGGTTTAGCCTGGACTTCTGTGG |  |
|  | CCCAACGCATCCTTGATGGTTTCTC |  |
|  | CTGAACGTATTGCAGGCCGAGTTGC |  |
